# A novel method to quantify IRDye800CW fluorescent antibody probes *ex vivo* in tissue distribution studies

**DOI:** 10.1186/2191-219X-2-50

**Published:** 2012-09-25

**Authors:** Sabrina Oliveira, Ruth Cohen, Marijke Stigter-van Walsum, Guus AMS van Dongen, Sjoerd G Elias, Paul J van Diest, Willem Mali, Paul MP van Bergen en Henegouwen

**Affiliations:** 1 Division of Cell Biology, Department of Biology, Faculty of Science, Utrecht University, Utrecht, 3584 CH, The Netherlands; 2 Department of Pathology, University Medical Center Utrecht, Utrecht, 3508 GA, The Netherlands; 3 Department of Otolaryngology/Head and Neck Surgery, VU University Medical Center, Amsterdam, 1007 MB, The Netherlands; 4 Julius Center for Health Sciences and Primary Care, University Medical Center Utrecht, Utrecht, 3508 GA, The Netherlands; 5 Department of Radiology, University Medical Center Utrecht, Utrecht, 3508 GA, The Netherlands

**Keywords:** Optical molecular imaging, Tracer quantification, Biodistribution studies, Antibodies, EGFR

## Abstract

**Background:**

We describe a new method for biodistribution studies with IRDye800CW fluorescent antibody probes. This method allows the quantification of the IRDye800CW fluorescent tracer in percentage of injected dose per gram of tissue (% ID/g), and it is herein compared to the generally used reference method that makes use of radioactivity.

**Methods:**

Cetuximab was conjugated to both the near-infrared fluorophore IRDye800CW and/or the positron emitter 89-zirconium, which was injected in nude mice bearing A431 human tumor xenografts. Positron emission tomography (PET) and optical imaging were performed 24 h post-injection (p.i.). For the biodistribution study, organs and tumors were collected 24 h p.i., and each of these was halved. One half was used for the determination of probe uptake by radioactivity measurement. The other half was homogenized, and the content of the fluorescent probe was determined by extrapolation from a calibration curve made with the injected probe.

**Results:**

Tumors were clearly visualized with both modalities, and the calculated tumor-to-normal tissue ratios were very similar for optical and PET imaging: 3.31 ± 1.09 and 3.15 ± 0.99, respectively. Although some variations were observed in *ex vivo* analyses, tumor uptake was within the same range for IRDye800CW and gamma ray quantification: 15.07 ± 3.66% ID/g and 13.92 ± 2.59% ID/g, respectively.

**Conclusions:**

The novel method for quantification of the optical tracer IRDye800CW gives similar results as the reference method of gamma ray quantification. This new method is considered very useful in the context of the preclinical development of IRDye800CW fluorescent probes for optical molecular imaging, likely contributing to the selection of lead compounds that are the most promising for clinical translation.

## Background

Recent developments in the field of near-infrared fluorescence, with new fluorophores and new instrumentations, have contributed to the growing interest in optical imaging [1-3] not only for noninvasive detection of tumors, but also in the context of image-guided surgery [4-6]. Thus far, clinical studies have been conducted with indocyanine green (ICG), used as ‘blood pool’ contrast agent, enabling the visualization of highly vascularized regions such as tumors or the detection of sentinel lymph nodes. ICG was, until very recently, the only near-infrared fluorophore approved for clinical use.

Importantly, the development of tumor-targeted probes for optical imaging has encouraged many researches in the last 10 years, leading to many recently published preclinical studies. In this context, one of the new near-infrared fluorophores widely employed is the IRDye800CW (here abbreviated to IR). Not only can this fluorophore be efficiently conjugated to antibodies, smaller antibody fragments, or nanoparticles [7-11], but more importantly, this fluorophore is already available at a good manufacturing practice-compliant production grade for clinical studies (since September 2010, LI-COR communication, [12]). In fact, the first clinical trial involving this fluorophore has just been initiated in The Netherlands, where IR is conjugated to the monoclonal antibody bevacizumab, an antibody that specifically binds VEGF (Trial: NL37479.042.11).

One of the aspects that will make a probe in preclinical development to be considered as a lead compound, with potential for translation into the clinic, is its capacity to accumulate specifically in the tumor, enabling the acquisition of images with good contrast. In the preclinical context, assessment of near-infrared probes' distribution in tumors and organs has been made either with intact organs *ex vivo* or through imaging of tissue sections, using the same systems as used for noninvasive imaging of mice [9,11]. This practice only gives a relative indication and qualitative assessment of the probes' tissue distribution. Ideally, one would prefer to have a method that allows for accurate quantification of the near-infrared probe in percentage of injected dose per gram of tissue (% ID/g), like it is commonly done with radiolabeled probes. However, this accurate quantification is actually not so simple due to the possibility of quenching of the fluorescence when fluorophores are present at a high concentration and also to the scattering of photons by tissue components.

Here, we describe a new method that circumvents the above-mentioned limitation in quantification through homogenization of the organs and dilution of the tissue lysates in order to infer in the linear range of fluorescence. Furthermore, to demonstrate the validity of this method, we have compared this method with the traditionally used gamma ray quantification of radioactive-labeled probes. For this, the monoclonal antibody cetuximab was used as a model and was conjugated to both IR and 89-zirconium (^89^Zr) forming a dual-labeled probe, i.e., ^89^Zr-cetuximab-IR. Cetuximab is a chimeric (mouse/human) monoclonal antibody that binds specifically to the epidermal growth factor receptor (EGFR), competing with ligand binding and promoting receptor internalization and downregulation [13]. Currently used in the clinic for cancer therapy, cetuximab has also been evaluated as a probe for molecular imaging, both at preclinical [14,15] and clinical levels (Prof. Guus van Dongen, personal communication).

In this study, in order to compare the two biodistribution methods, cetuximab is employed as a dual-labeled probe and intravenously injected in nude mice bearing A431 human tumor xenografts. After the collection of tumors and organs, each of these was divided into two pieces so that both methods could be employed for quantification of the probe in each organ.

## Methods

### Production of (dual) labeled probes: ^89^Zr-cetuximab-IR

Two different probes were prepared: an *imaging probe* and a *biodistribution probe.* The imaging probe is a mixture of a radioactive-labeled probe and fluorescently labeled probe, i.e., ^89^Zr-cetuximab + cetuximab-IR, while the biodistribution probe is a dual-labeled probe, i.e., ^89^Zr-cetuximab-IR. The monoclonal antibody (mAb) cetuximab (Erbitux; 5 mg/mL) was purchased from Merck (Darmstadt, Germany) and, before any chemical modification, cetuximab was buffer-exchanged on a PD10 column (GE Healthcare Life Sciences, Eindhoven, The Netherlands) to a solution of 0.9% NaCl. ^89^Zr (*t*1/2 = 78.4 h) was purchased from IBA Molecular (Louvain-la-Neuve, Belgium) as [^89^Zr]Zr-oxalate in 1.0 M oxalic acid (≥0.15 GBq/nmol) (http://www.iba-molecular.com/).

#### Conjugation of chelator and radiolabeling

Cetuximab was modified using the chelator desferal (Df; desferrioxamine B, Novartis Pharma BV, Arnhem, The Netherlands) and subsequently labeled with ^89^Zr as previously described by Cohen et al. [8]. The final concentration was 8.6 MBq/mg cetuximab for the biodistribution probe and 182 MBq/mg cetuximab for the imaging probe.

#### Conjugation of IRDye800CW

IRDye800CW-NHS ester (MW 1,166 Da, LI-COR Biosciences (Lincoln, NE, USA); herein designated as IR) was supplied by Westburg BV, Leusden, The Netherlands. For both the biodistribution probe and the imaging probe, the conjugation of IR was performed as previously described [8]. In short, ^89^Zr-cetuximab or cetuximab were brought to a pH of 8.5 by adding 0.1 M Na_2_CO_3_. Subsequently, 20 μL of IR diluted in dimethyl sulfoxide was added, and the total volume was adjusted to 1 mL with 0.9% NaCl. The IR was added to the mAb solution at a 2:1 molar ratio. The reaction mixture was incubated for 2 h at 35°C in a thermomixer at 550 rpm. The unreacted IR was removed by purification of the conjugates on a PD10 column, using 0.9% NaCl as eluent. The flow through and the first 1.5 mL were discarded. The next 2 mL containing the conjugated mAb was collected.

#### ITLC, HPLC, and SDS-PAGE analyses

The biodistribution probe and the imaging probe were analyzed by instant thin-layer chromatography (ITLC) for radiochemical purity, by high-performance liquid chromatography (HPLC) for mAb integrity and purity, and by sodium dodecyl phosphate polyacrylamide gel electrophoresis (SDS-PAGE) for purity of the probes. ITLC analysis was performed on silica gel-impregnated glass fiber sheets (PI Medical Diagnostic Equipment BV, Tijnje, The Netherlands), with a 20 mM citrate buffer having a pH of 5.0 as the mobile phase. HPLC analysis was performed on a JASCO Benelux BV HPLC (de Meern, The Netherlands) with a diode array detector system and an inline radiodetector (Raytest Isotopenmessgeräte GmbH, Straubenhardt, Germany) using a Superdex 200 10/300 GL size exclusion column (GE Healthcare Life Sciences). The eluent consisted of 0.05 M sodium phosphate, 0.15 M sodium chloride, plus 0.05% sodium azide (pH 6.8), and the flow was set at a rate of 0.5 mL/min. HPLC measurements were performed at *A* = 280 nm to measure cetuximab absorption, at *A* = 430 nm to measure the absorption of *N*-sucDf-Fe(III), and at *A* = 780 nm to measure the absorption of IR. The chelate-to-cetuximab and IR-to-cetuximab molar ratios were determined by HPLC, using the areas under the curve at *A*280, *A*430, and/or *A*780. Gel electrophoresis was performed as previously described [7,8]. In short, SDS-PAGE of ^89^Zr-cetuximab-IR was performed on a Phastgel system (GE Healthcare Life Sciences) using 7.5% polyacrylamide gels at nonreducing conditions, followed by phosphor imaging analysis. In parallel, cetuximab and ^89^Zr-cetuximab-IR were size-separated on a 15% polyacrylamide gel under reducing conditions. These proteins were then stained with the Coomassie Brilliant Blue solution (SERVA Electrophoresis GmbH, Heidelberg, Germany), and an Odyssey scanner (LI-COR Biosciences) was used for detection, employing the 800 nm detector for visualization of IR and the 700 nm detector for visualization of the Coomassie stain.

#### *In vivo* studies

Female athymic nude mice weighing 20 to 25 g and being 7 to 8 weeks of age (Harlan Nederland, Horst, The Netherlands) were housed in sterile cages under standard conditions (24°C, 60% relative humidity, 12-h light/dark cycles) and provided with water and food *ad libitum*. These studies were performed according to national regulations and approved by the local animal experiments ethical committee. Subcutaneous tumors were induced by inoculating two million cells of the A431 human epidermoid carcinoma cell line at the right and left hind legs. Approximately 1 week after tumor cell inoculation, the tumor sizes were 100 to 200 mm^3^, and the mice were randomly assigned to two different groups: (a) imaging group, consisting of four mice that were injected with the imaging probe, and (b) biodistribution study group, consisting of six mice that were injected with the biodistribution probe*.* All animal experiments were done according to the NIH Principles of Laboratory Animal Care and the Dutch national law (Wet op de dierproeven, Stb 1985, 336).

#### Optical imaging

During optical imaging, the mice were anesthetized with 2% isofluorane. Images were collected before and right after injection of the imaging probe and at 24 h post-injection (p.i.). Each image acquisition took less than 1 min, and images were obtained with two mice at a time, using the IVIS Lumina system with ICG filter sets (Caliper Life Science, Hopkinton, MA, USA). Data were analyzed with the living image software from Xenogen version 3.2 (Caliper LS).

#### PET imaging

Positron emission tomography (PET) imaging was performed on a HRRT PET scanner (Siemens/CTI, Munich, Germany [16]), a dedicated human brain scanner. The mice were anesthetized by inhalation of 2% isofluorane, and scanning time was 1 h. Transmission scans for attenuation and scatter correction were routinely obtained with each emission scan. Three-dimensional (3D) emission scans were acquired in list mode in 60 min. A single-frame static image was reconstructed using ordinary Poisson ordered subset expectation maximization. For visualization of the images, the freely available Amide's A Medical Imaging Data Examiner program was used [17].

### Biodistribution study

The mice were anesthetized, bled, euthanized, and dissected 24 h after injection of the dual-labeled ^89^Zr-cetuximab-IR, i.e., the biodistribution probe. Blood, tumors, and several organs were halved; each piece was weighed, and half was snap frozen for quantification of the IR, while the other half was immediately prepared for gamma quantification. No fixation of tissues was applied in any of these procedures.

#### Quantification of IR fluorescence

For IR fluorescence quantification, half of each organ and tumor was disrupted with a TissueLyser II system (Qiagen, Venlo, The Netherlands) using pre-cooled Eppendorf holders, 5-mm stainless steel beads, and RIPA buffer (50 mM Tris–HCl pH 7.4, 150 mM NaCl, 1 mM EDTA, 1% Triton X-100, 0.1% SDS) supplemented with a complete EDTA-free mini tablet protease inhibitor cocktail (Roche Applied Science, Penzberg, Germany). Then, homogenates were diluted in series (1:2 dilution steps) in 96-well plates, with the same buffer used for homogenization of the organs, which is done to determine the range where fluorescence intensity varies in a linear manner with the concentration. In parallel, the dilution series of the probe were also made using RIPA buffer for the same purpose. The intensity of the IR fluorescence of the samples and standard probe was detected at 800 nm with the Odyssey scanner. Subsequently, the concentration of the probe present in the homogenates was extrapolated from the calibration curves made with the standard probe, using the software Prism 5 (GraphPad Software Inc., La Jolla, California, USA). These concentration values were used to calculate the percentage of injected dose per gram of tissue (% ID/g), based on the volume of the homogenate and the weight of the tumors and organs.

#### Quantification of gamma rays

For gamma ray quantification and the evaluation of the biodistribution of ^89^Zr-cetuximab-IR, the other half of each organ and tumor was placed in 8.5 mL tubes (Sarstedt, Nümbrecht*,* Germany) with water, and the amount of radioactivity was measured in a γ-well counter (Wallac LKB-CompuGamma 1282; Pharmacia, Uppsala, Sweden). Radioactivity uptake was measured as % ID/g.

### Statistics

Biodistribution data are presented as mean ± SEM. The agreement between the results obtained with the two different quantification methods was evaluated by the Bland-Altman analysis including a linear regression approach [18,19]. For this, the average % ID/g of the ^89^Zr and IR quantifications for each sample is plotted - on a logarithmic scale in view of the observed range in % ID/g - against the difference in % ID/g of both measurements for each sample. To assess whether one of the two quantification methods consistently exceeds the other, the mean paired difference in % ID/g was calculated over all samples. Linear regression was then used to assess whether (1) the magnitude of this mean difference and (2) the variation between the two quantification measurements were related to the average % ID/g [19]. For the magnitude, the differences in % ID/g were regressed on the log-transformed average % ID/g values. As no statistical evidence was found about the magnitude of the between-method differences being related to the average % ID/g, the line of best agreement was simply defined as the overall mean paired difference in % ID/g over all samples. For the variation, the absolute residuals around the line of best agreement (as derived from the magnitude) were then regressed on the log-transformed average % ID/g values. Statistical evidence was found on the variation between the two quantification measurements which was related to the average % ID/g, with a good log-linear fit. Using the results from these linear regression analyses, the 95% limits of agreements between the two methods were then calculated [19]. The line of best agreement and the 95% limits of agreement are shown in the Bland-Altman plot. Statistical analyses were performed with the software Prism 5 (GraphPad Software Inc., La Jolla, California, USA) and SPSS Statistics 17.0 (SPSS inc., Chicago, Illinois, USA), with a two-sided cutoff for a statistical significance of 5%.

## Results

### Production and analysis of ^89^Zr-cetuximab-IR

To employ the new method for quantification of fluorescent probes in tissues and, at the same time, to enable the comparison with the reference method of gamma ray quantification, a dual-labeled probe was produced. The monoclonal antibody that binds to EGFR, i.e., cetuximab, was selected as a model system, and it was conjugated to IR and/or radiolabeled with ^89^Zr. As shown previously [8], this dual-labeling protocol is possible in a manner that does not affect the binding properties of cetuximab to EGFR nor the biodistribution of the probe. Two probes were produced in this study: the first probe was particularly prepared for the biodistribution study, consisting of cetuximab which was dually conjugated to the radioactive and fluorescent label (i.e., dual-labeled biodistribution probe: ^89^Zr-cetuximab-IR); and the second probe was dedicated for imaging and consisted of a mixture of radioactive-labeled cetuximab and fluorescently labeled cetuximab (i.e., imaging probe: ^89^Zr-cetuximab + cetuximab-IR). The differences are in the radioactive dose, as PET imaging requires a higher dose than the biodistribution studies.

Analysis of the biodistribution probe revealed that, on average, the 0.5 group of the chelator Df was coupled to cetuximab and the radiolabeling with ^89^Zr resulted in an overall labeling yield of 70%. ITLC and HPLC showed that the radiochemical purity of the product always exceeded 95% after purification on PD10. The conjugation efficiency of IR to ^89^Zr-cetuximab was approximately 50%, resulting in 0.9 groups of IR per molecule of ^89^Zr-cetuximab-IR as assessed by HPLC analysis. After purification on PD10, the dual-labeled cetuximab was found to be more than 99% pure for ^89^Zr as well as for IR. For the imaging probe, the conjugation efficiency of IR to cetuximab was approximately 70%, resulting in 1.4 groups of IR per molecule cetuximab. The purity of the probes produced as well as the successful conjugation of ^89^Zr and IR was also confirmed by gel electrophoresis, highlighting the reproducibility of the protocols employed.

Previous experiments have shown that the immunoreactivity of ^89^Zr-cetuximab-IR is 99% at infinite antigen access studies [8]. Similarly, ^89^Zr-cetuximab-IR was found to be stable when stored in 0.9% NaCl at 4°C for at least 4 days, without any loss of integrity and immunoreactivity as assessed by HPLC or binding assays. Moreover, only a minimal percentage of IR was found to be released from the antibody when stored in human serum, i.e., approximately 1.4% [8]. Overall, the produced fluorescent and radioactive probe fulfills the requirements needed for the following studies.

### Optical and PET imaging

**Figure 1:**
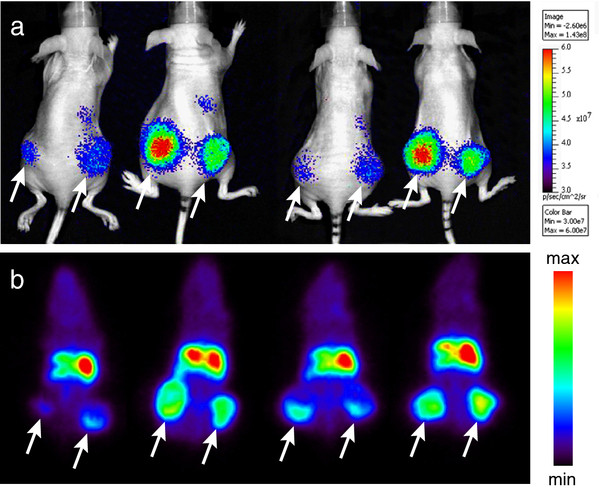
**Mice imaged with optical and PET modalities.** Nude mice bearing A431 human tumor xenografts at the hind legs were imaged 24 h post-injection of the imaging probe, i.e., ^89^Zr-cetuximab + cetuximab-IR. (**a**) Optical images taken with IVIS Lumina at 1-s exposure time and ICG filter sets. (**b**) PET images representative of the entire scan (1 h), coronal view. Tumors are indicated with arrows. Individual tumor weights determined at the end of the experiment, from left (L) to right (R), are as follows: mouse 1: L 0.2544 g, R 0.4353 g; mouse 2: L 0.4176 g, R 0.243 g; mouse 3: L 0.529 g, R 0.2163 g; mouse 4: L 0.5474 g, R 0.2972 g.

To observe the distribution of the EGFR-targeted probe with the two imaging modalities, the nude mice bearing A431 human tumor xenografts at the hind legs were injected with the imaging probe. A group of four mice was injected with 170 μL containing 38.3 μg of ^89^Zr-cetuximab mixed with 62.72 μg of cetuximab-IR. At 24 h after injection of the probe, these mice were imaged with an optical imager and a PET scanner. Both imaging modalities allowed a very clear delineation of the tumors (Figure 1). The abdominal area is also well visible, corresponding mainly to the liver. From the images collected and the registry of the individual weight of tumors at the end of the experiment, a direct correlation is observed between tumor weight and signal intensity which accumulated at the tumors. Based on these images, tumor-to-normal tissue (T/N) ratios were calculated by drawing regions of interest around the tumors and in normal tissues (namely at the head of the mice), for background reference. The T/N ratios obtained were 3.31 ± 1.09 for the optical images and 3.15 ± 0.99 for the PET images. Overall, a similar probe distribution was observed in the different images obtained with the two imaging modalities.

### Biodistribution assessed by IR fluorescence as well as gamma ray quantifications

In order to apply both methods for assessment of the biodistribution, i.e., the quantification of IR fluorescence and the reference method of gamma ray quantification, the following setup was employed: a group of six mice was injected with 120 μL containing 106 μg of ^89^Zr-cetuximab-IR, i.e., the biodistribution probe. Twenty four hours later, each mouse was anesthetized, bled, euthanized, and dissected, and each organ collected was halved and weighed. One half of each organ was processed for IR fluorescence quantification, and the other half was processed for gamma ray quantification.

**Figure 2:**
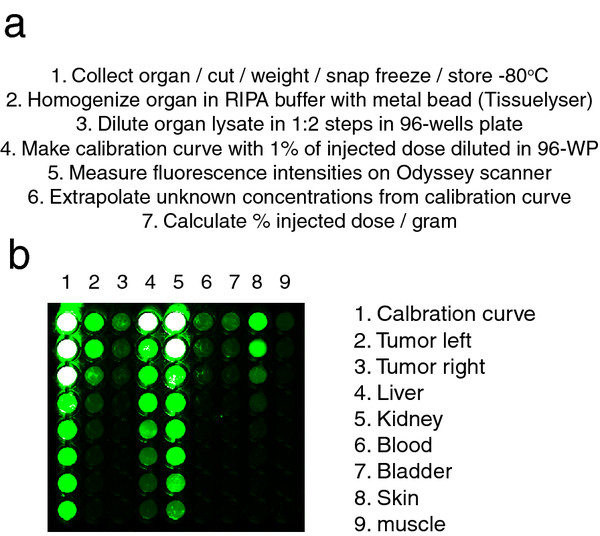
**New method for quantification of IR fluorescence in tissues.** (**a**) Overview of the method step-by-step. (**b**) Example of an image obtained with the Odyssey scanner while measuring IR fluorescence (shown in green) intensities from the calibration curve and lysates of organs, all diluted in 1:2 steps in a 96-well plate (from top to bottom). Saturation of the fluorescence is shown in white.

Quantification of gamma rays was performed using established methods as described in the ‘Methods’ section. The procedure for quantification of IR fluorescence is depicted in Figure 2. In brief, the tumors and organs were first homogenized and then diluted several times in 96-well plates. These plates were imaged with an Odyssey scanner to measure the IR fluorescence intensities, and at the same time, the biodistribution probe was also diluted in order to make a calibration curve of concentration versus fluorescence intensity (Figure 2). Especially in organs with the highest accumulation of the fluorescent probe, saturation of the fluorescent signal can occur, which will, without the dilution approach, result in an underestimation of the signal in the particular organ (Figure 2b). From the obtained curve, the unknown values of fluorescence of the diluted samples were extrapolated, taking only the values of the dilutions where linear range of fluorescence intensity was detected. These concentration values, together with the initial weight of the tumor or organ and the volume of lysates, were then used to calculate the % ID/g values.

**Figure 3:**
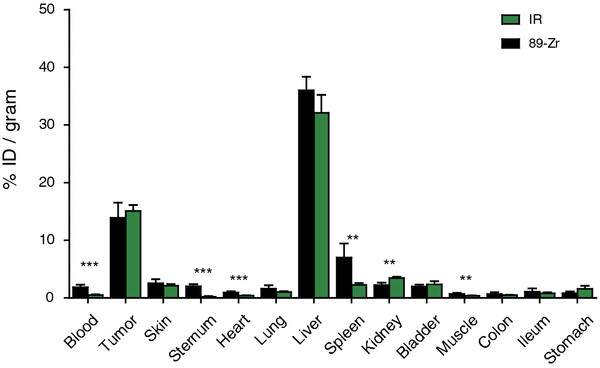
**Biodistribution of**^**89**^**Zr-cetuximab-IR in tissues assessed by IR fluorescence and gamma ray quantifications.** Tumors and organs were collected 24 h p.i. of the biodistribution probe. Each organ was halved: one half was processed for IR fluorescence quantification and the other half for gamma ray quantification. Six mice with two tumors each were employed; graph bars show mean values ± SEM. Statistical significance is as follows: double asterisks for *p* < 0.01 and triple asterisks for *p* < 0.001.

Both quantification methods led to comparable results in % ID/g and, thus, comparable biodistributions (Figure 3). In fact, for organs such as the tumor, liver, lung, and skin, no significant differences in uptake were obtained. Nevertheless, some differences obtained were statistically significant, namely, the quantification of probe in the blood, sternum, heart, spleen, kidney, and muscle. These differences might be related to the catabolism of the probe and/or the different retentions of the individual tracers, i.e., ^89^Zr and IR.

**Figure 4:**
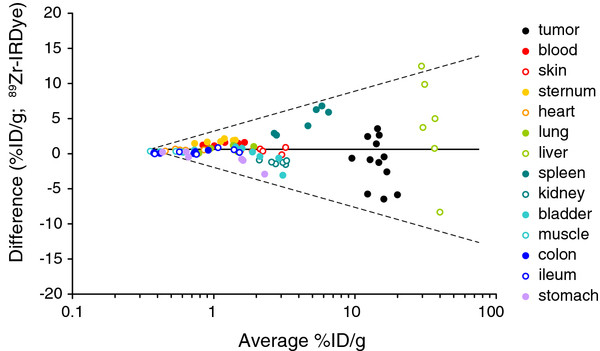
**Bland-Altman plot of IR quantification and gamma ray quantification for probe content in tissues.** Solid line denotes the line of best agreement (average difference ^89^Zr-IRDye, 0.63% ID/g of tissue); dashed lines denote 95% limits of agreement.

To compare the two sets of data, i.e., data obtained from IR fluorescence quantification and from gamma ray quantification, the Bland-Altman method of analysis was employed. Most importantly, the values obtained through gamma ray quantification are, on average, 0.63% ID/g of tissue higher than those of IR fluorescence quantification. This small average difference does not depend on the level of % ID/g itself (i.e., the average difference between the two methods does not show a statistically significant increase or decrease with increasing levels of % ID/g, as assessed by linear regression analysis), and could thus be solved by simple calibration. Furthermore, the Bland-Altman plot shows that the absolute difference in the values obtained with the two methods for matching samples (as each organ was split in two) is very small for samples with a small average % ID/g of tissue and that the variations between the two measurements increase log linearly with higher average values (as shown by the 95% limits of agreement indicated by the dashed lines in Figure 4).

## Discussion

During the last decade, many preclinical studies have been evaluating new near-infrared fluorescent probes for targeted molecular imaging, and the trend is to slowly move towards the translation into clinical practice. However, before this can be done successfully, a thorough and precise evaluation of the probe (or probes) is necessary at the preclinical level. In this context, the precise quantification of probes in tissues is a matter of concern in biodistribution studies. The approach most commonly used for assessment of tissue distribution of a probe consists of imaging of organs or sections of organs with an optical imager, only allowing for a relative or qualitative assessment. This makes comparison between different studies and different probes rather difficult, since no % ID/g value is possible to be obtained in this manner. This fact is related to the nature of fluorophores: their close proximity can result in quenching of the fluorescence, leading to an underestimation of the amount of probe in tissues. Also, the scattering of photons by tissue components can compromise the fluorescence detected, and moreover, the linear range of fluorescence detection is restricted.

Here, we describe a new method for the quantification of IRDye800CW fluorescent probes in tissues. This method circumvents the issues mentioned above by diluting the lysate of the homogenized organs in order to infer in the linear range of fluorescence (Figure 2). To validate our new method, we have compared it with the most commonly used method for biodistribution studies, i.e., gamma ray quantification of radiolabeled probes. To do so, and here as a research tool, a dual-labeled probe was prepared, minimizing the possibility that the tissue distribution of the probe would be affected by the different labels. The monoclonal antibody cetuximab was selected for this study as it was previously shown that certain conditions of dual labeling do not affect its biodistribution [8]. This could not be the case for smaller molecules or smaller antibody fragments due to the reduced size. Hence, for the biodistribution study, cetuximab was conjugated to the IRDye800CW fluorophore (IR) and to the positron emitter 89-zirconium (^89^Zr), forming the dual-labeled ^89^Zr-cetuximab-IR, i.e., the biodistribution probe. To further minimize possible variations, each organ and tumor that was collected for the biodistribution study was halved so that one half could be processed for IR fluorescence quantification and the other half for gamma ray quantification. Nevertheless, one has to bear in mind the possibility of heterogeneous uptake in each tumor and organ, which could lead to differences in uptake levels between the fluorescence and the radioactivity method.

Importantly, the results obtained with the two different methods are very similar for tumors and organs such as the liver, lung, stomach, skin, and intestines (Figure 3). Significant differences were obtained for the blood, heart, spleen, sternum, and muscle, showing higher values by gamma ray quantification, and the kidney which gave a higher value with IR fluorescence quantification. As control studies have shown the stability of the probe in serum, and as it is known that both ^89^Zr and IR residualize after receptor-mediated internalization of cetuximab ([20] and Dr. Mike Olive, personal communication), these variations are most likely related to what happens to the probe after liver catabolism. In fact, degradation of ^89^Zr-cetuximab-IR into small peptides is to be expected, and the fate of these fragments after excretion into the bile may vary, for instance, some of these might be more effectively reabsorbed into the bloodstream at the intestines and subsequently distribute differently throughout the tissues. Even though this is unclear, the trend suggested by the results presented here is that IR fragments are more efficiently eliminated by kidney filtration, and ^89^Zr-fragments accumulate in bone tissues like sternum, which is in agreement with other studies [12,20-22].

It is worth realizing that true values of % ID/g are unknown and that two groups of values have been obtained, corresponding to the quantification of IR fluorescence and gamma rays in the tissues. The aim of this study was to determine whether comparable information could be obtained with both quantification methods. In this context, Bland-Altman plots are used to compare the two sets of data and to determine how much each data set differs from the mean value of the two sets of data. Our results show a good average agreement between the data sets. Although the ^89^Zr measurements are, on average, slightly higher, this small average difference does not depend on the level of % ID/g itself and may thus be easily solved by a calibration factor. The disagreement between the two methods increases with higher values of % ID/g, e.g., in the spleen, tumor, and liver (Figure 4). This observation could simply be explained by a larger intra-organ variation in the case of the spleen, liver, and the tumor. It also shows that for between-group comparisons (e.g., a comparison between the uptake of two probes), more subjects will probably be needed to be able to detect a certain absolute average difference between those groups, when the uptake is, on average, high compared to low.

Overall, the results obtained through IR fluorescence quantification are considered to be representative of the results obtained with the reference method employed for radiolabeled probes. This was also suggested initially by the images obtained with the optical imager and the PET scanner (Figure 1). In that part of the study, a slightly different probe was employed (i.e., ^89^Zr-cetuximab + cetuximab-IR, the imaging probe), but in fact, no differences were to be expected concerning the biodistribution of these probes, as it has been previously investigated that the conditions here employed for coupling of IR or ^89^Zr are inert to cetuximab [8].

The optical images presented (Figure 1a) show a relatively weaker signal at the tumors and livers compared to the PET images (Figure 1b), but this is mostly related to the modality employed. The optical imager employed does not allow 3D collection of data, and fluorescence is only detected at the surface, whereas the PET scanner allowed for 3D collection of data. Recently, newer optical imagers have been developed employing the so-called fluorescence molecular tomography that is suggested to be able to quantify proteins or probes deep in tissues [23]. Nevertheless, background concentrations and tumor-to-normal tissue ratios have been reported as limiting factors [24], thus leaving room for the new method here described to be used for accurate quantification of IR fluorescent probes. In fact, this method will most likely be applicable to other near-infrared fluorescent probes, although residualization of the fluorophore should be confirmed as well as the stability of the probe in serum, being therefore advisable to be careful in generalizing this method to other fluorescent probes.

Even though the method for IR quantification is more laborious than gamma ray quantification, this new method enables accurate quantification of the probe in % ID/g without the use of radionuclides. This fact is indeed relevant when the probe under development is meant for optical imaging and not for PET or SPECT imaging. We have successfully applied this new method for the quantification of IR fluorescent anti-EGFR nanobodies or VHHs in tissues [7]. In this study, we were able to demonstrate that the small 15 kDa fluorescent nanobodies accumulate more rapidly and to a greater extent in A431 xenografts than the 150 kDa monoclonal antibody cetuximab.

## Conclusions

Here, we have presented a new method for *ex vivo* quantification of IR fluorescence in tissues, which gives comparable results to the reference method used for radiolabeled probes. Taking into account the current trend, in optical imaging, of moving towards the translation of targeted fluorescent probes to clinical diagnostic use, this new method is considered very valuable for preclinical assessment of tissue distribution of IR fluorescent probes, with an accuracy that thus far was inexistent. Thus, this new method can contribute to the selection of lead compounds that are most promising for clinical translation into probes for molecular imaging.

## Competing interests

The authors declare that they have no competing interests.

## Authors’ contributions

SO, GAMSvD, and PMPvBeH designed the experiments. SO, RC, and MSvW performed the experiments. SO and SGE performed the data analysis, and SO, GAMSvD, and PMPvBeH have written the manuscript. PvD and WM participated in important discussions. All authors have read and approved the final manuscript.
